# Norovirus waterborne outbreak in Chalkidiki, Greece, 2015: detection of GI.P2_GI.2 and GII.P16_GII.13 unusual strains

**DOI:** 10.1017/S0950268819000852

**Published:** 2019-06-27

**Authors:** K. Tryfinopoulou, M. Kyritsi, K. Mellou, F. Kolokythopoulou, V.A. Mouchtouri, M. Potamiti-Komi, A. Lamprou, Th. Georgakopoulou, C. Hadjichristodoulou

**Affiliations:** 1 Hellenic Centre for Diseases Control and Prevention, Department of Surveillance and Intervention, Athens, Greece; 2 European Public Health Microbiology Training Programme (EUPHEM), European Centre For Disease Prevention and Control, Stockholm, Sweden; 3 Department of Hygiene and Epidemiology, Medical School, University of Thessaly, Larissa, Greece

**Keywords:** Norovirus, outbreak, possibly-waterborne, recombinant strain, touristic region

## Abstract

Noroviruses, along with rotaviruses, are among the leading causes of gastroenteritis worldwide and novel strains are periodically emerging. In August 2015, an unusual increase of gastroenteritis cases occurred in a touristic district in Kassandra peninsula, Chalkidiki, Northern Greece. Seven stool specimens from cases were tested positive for norovirus. Molecular investigation and phylogenetic analysis identified that there was co-circulation of norovirus GI.P2_GI.2 and the recombinant strain GII.P16_GII.13. A 1:1 case–control study conducted and showed that tap water consumption significantly associated with developing symptoms of gastroenteritis (odds ratio = 36.9, *P* = 0.018). The results of the epidemiological investigation, the co-circulation of two different norovirus strains, the information of a pipeline breakage at the water supply system before the onset of cases, and reports on flooded wells and sewage overflow, indicated the possibility of water contamination by sewage during the pipeline breakage leading to a large outbreak with a peak at 10 August and a possible secondary person-to-person transmission after the 16th of August. Norovirus GI.P2_GI.2 strains are rarely reported in Europe, while it is the first time that infection from the recombinant strain GII.P16_GII.13 is recorded in Greece.

## Introduction

Noroviruses, along with rotaviruses, are the leading causes of acute non-bacterial gastroenteritis worldwide. Noroviruses affect all age groups, with higher incidence rates and more severe outcomes among young children and the elderly [1]. Noroviruses are non-enveloped positive single-strand RNA viruses and belong to the Caliciviridae family. They possess a 7.5–7.7 kb genome which consists of three open reading frames (ORFs) [2]. ORF1 encodes non-structural proteins including the RNA-dependent RNA-polymerase (RdRp), ORF2 encodes the major capsid protein VP1 while the ORF3 encodes the minor capsid protein VP2. To date, noroviruses are classified into six genogroups (GI–GVI) based on the diversity of the VP1 gene [2]. Each genogroup can be subdivided into genotypes; eight genotypes of GI, 19 genotypes of GII, two genotypes of GIII and one for GIV, GV and GVI have been recorded so far, based on either RdRp or VP1 sequences [2].

Noroviruses demonstrate high-genetic variability rates, which are mainly attributed to point mutations, antigenic drift and recombination events [3]. Recombination events are regarded as the main mechanism for norovirus evolution and are usually located between the RdRp and capsid gene near or within the ORF1/ORF2 overlapping region. Thus, genotyping both sequences is essential in order to detect new recombinant variants [2]. Over the last decade, GII.4 was the most common genotype circulating worldwide and recombinant GII.4 strains have been recorded [4, 5] whereas numerous recombinant strains have also been reported within GI, GII and GIII [3–9]. The primary route of norovirus transmission is the faecal–oral either by direct person-to-person spread or by ingestion of contaminated water or food [10]. Due to their environmental persistence, their low infectious dose (10–100 viral particles can induce symptoms), the short-time immunity and naturally occurring norovirus recombinants, noroviruses have potential to cause large outbreaks.

Large foodborne and waterborne norovirus outbreaks have been widely documented in the literature and have been increasingly identified in Europe and elsewhere [7, 11, 12]. The increased detection of norovirus outbreaks can be attributed to the development of molecular methods and the increasing use of real-time reverse transcriptase-polymerase chain reaction (RT-PCR), which has improved both the sensitivity and the specificity of norovirus laboratory testing methods [13]. Evidence shows the importance of early detection of outbreaks via an organised surveillance system even in light of accurate diagnostic methods. In Greece, norovirus outbreaks have been increasingly reported the last years (Hellenic Centre for Disease Control and prevention (HCDCP), unpublished data), however the real impact of norovirus infections in Greece is not very well understood, since comprehensive surveillance data on viral gastroenteritis infections are currently lacking.

Here we report the findings of an outbreak of norovirus gastroenteritis, possibly associated with drinking water from a municipal supply network, in a touristic area during the high season in Chalkidiki, northern Greece. The objectives of the investigation were the assessment of the extent of the outbreak, the identification of the mode and vehicle of transmission as well as of possible risk factors in order to implement appropriate measures to control the outbreak.

## Methods

### Epidemiological investigation

On 11 August 2015, the Hellenic Centre for Disease Control and prevention was informed by the Head of the primary Health Care Centre (HCC) of Kassandra in Chalkidiki, North Greece about an increased number of cases with gastrointestinal symptoms (mainly vomiting and diarrhoea) since 9th of August, mainly from a specific municipal district consisting of a main village and its coastal settlement about 2 km apart. According to the population census of 2011, the municipal district has 1079 permanent residents (543 living at the main village and 536 living at the coastal settlement). According to the available data from the national mandatory notification system for clusters or outbreaks of gastroenteritis as well as the records of the Health care Centre, only small gastroenteritis clusters had been identified in this region since 2008. On the same day of the alert we asked from the personnel of the HCC to fill in a structured form (line-listing) with the descriptive data and the contact details of all cases with gastroenteritis symptoms that visited the HCC since the beginning of August. We also requested further daily reporting. The main hypothesis was of a common-source gastroenteritis outbreak. Initial telephone interviews with several cases using trawling questionnaires did not conclude to any common activities, meals or other exposures. An analytical epidemiological study was decided as until then, there was no laboratory confirmation of the causative agent to the gastroenteritis outbreak.

A 1:1 case–control study was conducted. Based on the collected descriptive data, a case was defined as a person with vomiting and/or diarrhoea who had been in the main village and/or the coastal settlement (resident or tourist) between 9 August and 13 August 2015. We reviewed the medical records of the HCC and we selected all cases that fulfilled the case definition ensuring that one case per family was included in the study. We defined control as any person staying at the main village and/or the coastal settlement (resident or tourist) from 9–13 of August without presenting symptoms of vomiting and/or diarrhoea. We actively searched for controls using the local telephone directory through random digit-dialling and interviewed both cases and controls over telephone. We collected information via a structured questionnaire which included information on demographic characteristics, the exact residence address, clinical manifestations and severity of the disease, participation in social activities/events, food and water consumption as well as questions on a number of proxies for exposure to tap water. In order to differentiate secondary cases we asked about prior contact with symptomatic cases. All questions concerned the 2 days prior to the disease onset, while controls were asked about the same exposures in the 2-day period prior to 10 August, which was the day with the highest number of recorded cases based on the onset of symptoms.

In order to have a broader estimation of the attack rate in the community, we searched for cases that did not visit the HCC by contacting a list of all hotels and other accommodation. We contacted the persons in charge, asking for any clients with gastroenteritis symptoms from the beginning of August, and if there were any, the exact number of them as well as the number of clients during the aforementioned period.

### Laboratory investigation

#### Clinical samples

Stool samples from patients that visited the HCC were tested at the Regional Public Health Laboratory of Thessaly for pathogenic bacteria (*Salmonella* spp., *Shigella* spp., *Campylobacter* spp., *Yersinia* spp., *Escherichia coli* O157:H7) by standard culture methods.

Molecular analysis for rotavirus and norovirus detection was performed. For the nucleic acid extraction the iPrep™ Invitrogen Purification Instrument (Thermo Fisher Scientific) with the iPrep PureLink Invitrogen Virus Kit (Thermo Fisher Scientific) was used. A RT-PCR was performed using the Super Script III One-Step qRT-PCR System with Platinum Taq Polymerase kit (Invitrogen), in a validated Eppendorf Mastercycler Gradient System, targeting norovirus and rotavirus separately. Master mixes (45 µl) with primer concentrations of 10 mol/μl were prepared and amplified for each virus respectively. The primers used were JV12Y and JV13I for norovirus amplification, and Beg9band End9bfor Rotavirus amplification (the primers used are available upon request).

Samples positive for norovirus detection were sequenced following a single protocol as it was described by van Beek *et al*. [8]. This protocol enabled the amplification of a 1000 bp fragment of the ORF1/ORF2 overlapping region in the same amplicon, eliminating the risk that sequences from polymerase and capsid genes from different norovirus strains were present in the same sample. Moreover, genotyping regions of both the RdRp and capsid gene could reveal potential recombination events near or within this region. The usual recombination point in ORF1/ORF2, from nucleotides 4523 to 5277 and from nucleotides 4527 to 5162 was covered (primers and the RT-PCR conditions used are available upon request).

Sequence identity was determined using both RIVM Noronet, Norovirus Genotyping Tool version 2.0 (https://www.rivm.nl/mpf/typingtool/norovirus/) and BLAST (https://blast.ncbi.nlm.nih.gov/Blast.cgi).

#### Environmental samples

Water samples from different points of the water supply network of both the main village and the coastal settlement as well as from a drilling (ground water well) from the coastal settlement were collected and analysed by a local private laboratory according to ISO methods on 11 August 2015 and 20 August 2015. Samples were subjected to standard microbiological analysis for coliform bacteria and *E. coli* (ISO 9308-01:2000), intestinal enterococci (ISO 7899-02:2000) and total aerobic count at 22 and at 37 °C (ISO 6222:1999). The samples were also analysed for basic chemical parameters (pH, conductivity, nitrites, nitrates, chlorine residue). No virological testing was performed. The water samples microbiological quality was assessed according to the parameters in the national legislation for water intended for human consumption (ΚΥΑ Υ2/38295/2007 ΦΕΚ 630/26-4-2007).

### Environmental investigation

Information about possible problems of the water supply network and the sewage drainage system before the emergence of the outbreak, as well as mapping of the water distribution pipeline in the affected district, were requested from the municipality.

### Ethical

HCDCP is authorised to process personal data during outbreaks, by the Greek law. The study protocol was submitted to the ethical committee of the National School of Public Health for approval. The participants of the study were interviewed over phone by health professionals of HCDCP and the data analysis was processed by the main researcher. Data were used only for the purposes of the given investigation. During telephone interviews participants were informed on the scope of the study and that they could refuse to participate.

### Statistical analysis

A database was created with the use of EpiData 3.1 software. SPSS v. 20.0 (SPSS Inc., USA) was used for data analysis. Contingency tables (univariate analysis) with the calculation of *χ*^2^ test or Fisher's exact test were used for the evaluation of the associations between the categorical variables. Associations between quantitative variables were tested with the Student's *t* test (for normally distributed variables), after checking for homogeneity of variance, or the Mann–Whitney test for skewed variables. The odds between exposed and non-exposed were compared, and odds ratios (OR) and the respective 95% confidence intervals (CIs) were calculated. An association was considered statistically significant when *P* ⩽ 0.05; all calculated *P*-values were two-tailed. Since the water supply zone could be a significant modifier of the association between the disease occurrence and consumption of tap water (or other exposures), a stratified analysis was performed. ORs for the two strata (the coastal settlement water supply system and the rural water supply system at the village) were calculated and compared for the identification of effect modification. Multivariable analysis was restricted to the stratum ‘coastal settlement’. All statistically significant factors in the univariate analysis were included in the multivariable analysis.

## Results

### Epidemiological investigation

In total, 230 gastroenteritis cases were recorded at the HCC from 9 August to 28 August 2015. Among the 230 recorded cases, 108 (47%) were localised at the specific municipal district. The remaining 122 recorded cases were distributed in the 13 neighbouring municipal districts (small villages) with a range of 1–3 cases per day, which was considered as expected for the villages' populations during summertime.

**Fig. 1.**
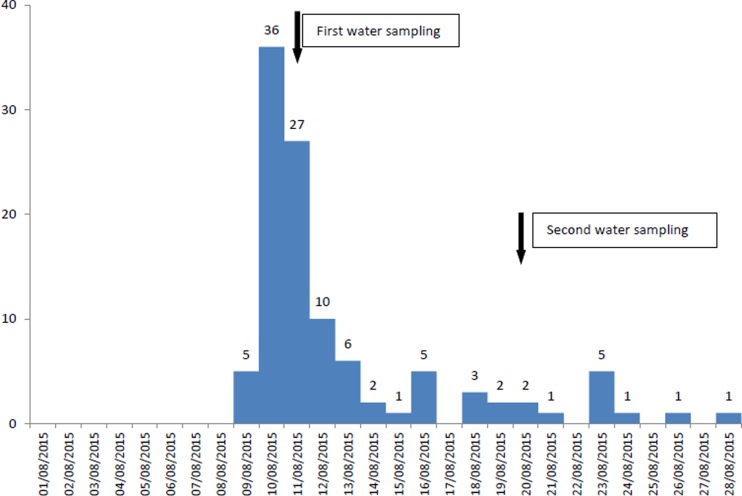
Number of recorded cases (*n* = 108) by date of symptoms onset and water sampling, 9–28 August 2015, municipal district of the main village and its coastal settlement, Kassandra peninsula, Chalkidiki.

In the municipal district, where the number of recorded cases (*n* = 108) was much higher than expected, the cases increased on 9 August, peaked on 10 August and substantially decreased on 13 August, whereas since 16 August, a smaller increase of cases was reported (possible secondary transmission) (Fig. 1). Cases represented all ages (range 1–82 years old), while 60 (56%) were female. Eighty-six recorded cases were Greek tourists (79.6%), while no foreign tourists were recorded at the HCC.

A total of 84 people fulfilled the criteria for being a case for the analytical study. Among them, for 15 cases there was no telephone or other contact details available, seven were relatives of another case (same household), eight cases could not be reached despite the repeated calls during different days and time and finally two refused to participate. We finally interviewed 52 cases. Eleven cases reported previous contact with another case and were excluded as possible secondary cases. In total, 41 cases and 50 controls were included in the case–control study. Age, gender distribution and residence details are presented in Table 1. The mean (±s.d.) age of cases was 32.2 ± 20.8 years. The majority of cases were Greek tourists or seasonal residents (owners of summerhouses) (85.4%) residing in the coastal settlement (80.6%) while the remaining were locals.

**Table 1.** Results of univariate analysis, tap water consumption and tap water consumption proxies, Kassandra peninsula, Chalkidiki, Greece, 2015

Characteristic/exposure	Cases	Controls	Crude OR	95% CI	*P*-value
Age (mean, ±s.d.), years	32.2 ± 20.8	51.6 ± 16.0			<0.001
Sex (male)	22/41	33/50	0.59	0.26–1.39	0.230
Residence place (coastal settlement strictly)	33/41	28/50	3.24	1.25–8.41	**0.013**
Social event	7/41	16/50	0.44	0.16–1.20	0.103
Meal/drink outside home	30/41	16/50	5.80	2.33–14.42	**<0.001**
Outdoor activities in Skala	34/41	20/50	7.28	2.71–19.63	**<0.001**
Consumption of tap water at home/outside	30/41	18/50	4.84	1.97–11.93	**<0.001**
Drinks with icecubes outside home	26/31	16/40	7.80	2.48–24.57	**<0.001**
Tap water for washing fruits and vegetables	37/41	46/50	0.80	0.19–3.44	0.768
Tap water for brushing teeth	41/41	46/50	n.a.	n.a.	0.064
Tap water for making ice cubes	32/41	40/49	0.80	0.28–2.25	0.670
Use of dishwasher	11/41	19/50	0.59	0.24–1.46	0.259
Use of filter	10/41	11/50	1.14	0.43–3.04	0.780

n.a.: not-applicable.

The reported symptoms among cases were vomiting (85.4%), diarrhoea (75.6%), fever (defined as body temperature >38 °C) (51.2%), abdominal pain (41.5%), nausea (31.7%) and fatigue (22%). The median reported duration of symptoms was 2 days (range 0.5–7 days).

According to the results of the univariate analysis (Table 1), the disease occurrence was associated with staying/living at the coastal settlement (OR 3.24, 95% CI 1.25–8.40), having meal/drink outside home (OR 5.8, 95% CI 2.33–14.41), having outdoor activities in the coastal settlement (OR 7.28, 95% CI 2.71–19.41), consuming tap water (OR 4.84, 95% CI 1.97–11.93) and having drinks with ice cubes (OR 7.8, 95% CI 2.48–24.57). No food item consumption was related to disease occurrence.

Furthermore, we performed a stratified analysis, based on the information that the main village and the coastal settlement have the same central tank, but different main and secondary pipelines for water supply. The results of stratified analysis are shown in Table 2. The place of residence was a modifier of the association between the disease occurrence and the consumption of tap water.

**Table 2.** Univariate and stratified analysis, tap water consumption and tap water consumption proxies, Kassandra peninsula, Chalkidiki, Greece, 2015

Exposure	Cases (*n* = 41)	Controls (*n* = 50)	Crude OR	95% CI	OR, coastal settlement	95% CI	OR, main village	95% CI
Age (mean, ±s.d.), years	32.2 ± 20.8	51.6 ± 16.0						
Sex (male)	22/41	33/50	0.59	0.26–1.39	0.71	0.26–1.94	0.88	0.13–5.82
Residence place (coastal settlement)	33/41	28/50	3.24	1.25–8.41	n.a.		n.a.	
Meal/drink outside home	30/41	16/50	5.80	2.33–14.42	6.90	2.22–21.40		
Outdoor activities in Skala	34/41	20/50	7.28	2.71–19.63	6.69	2.14–20.85	n.a.	
Consumption of tap water at home/outside	30/41	18/50	4.84	1.97–11.93	7.33	2.31–23.32	n.a.	
Drinks with icecubes outside home	26/31	16/40	7.8	2.48–24.57	14.38	3.28–63.01	1.69	0.22–12.81
Tap water for washing fruits and vegetables	37/41	46/50	0.8	0.19–3.44	0.56	0.09–3.30	n.a.	
Tap water for brushing teeth	41/41	46/50	n.a.		n.a.		n.a.	
Use of dishwasher	11/41	19/50	0.59	0.24–1.46	1.61	0.42–6.22	n.a.	
Use of filter	10/41	11/50	1.14	0.43–3.04	0.99		2.04	0.36–11.67

n.a.: not-applicable.

As among the residents of the coastal settlement, gastroenteritis occurrence was associated with having drinks with ice cubes (OR = 14.4, 95% CI 3.28–63.0), consumption of tap water (OR = 7.33, 95% CI 2.31–23.32), having meal/drinks outside home (OR = 6.9, 95% CI 2.22–21.42) and participating in outdoor activities (OR = 6.69, 95% CI 2.14–20.58), we performed a multivariable analysis for stratum coastal settlement. In the conditional logistic regression model, among residents of the coastal settlement the only variable significantly associated with the disease occurrence was drinking tap water (OR = 36.9, *P* = 0.018).

Among the 41 primary cases included in the analytical study, six reported one more case and 21 reported more than one cases in their household (range 2–5 cases). Thus, the disease burden in the community was considered to be high, taking into account also that foreign tourists, mainly from Serbia and Russia did not attend the HCC as they had their own group doctors for health insurance reasons according to hotel owners. Managers of hotels located nearby the central square of the coastal settlement reported an almost 100% attack rate among their clients, whereas the remaining hotels located in a distance from the central square reported few sporadic cases.

### Laboratory investigation

#### Clinical samples

Stool samples were collected from seven cases, four male and three female. The samples were positive for norovirus detection and negative for *Salmonella* spp., *Shigella* spp., *Campylobacter* spp., *Yersinia* spp., *E. coli* O157:H7 and rotavirus. The median age of laboratory confirmed norovirus gastroenteritis cases was 28 years, with a range of 2–75 years old. Four of them were residents or tourists in the coastal settlement, two were owners of summer houses located between the main village and the coastal settlement, and for the remaining one there was no residence status data available.

Of the seven positive for norovirus stool samples, three were sequenced in order to determine the genogroup and genotype of the strains. Both RIVM Noronet, Norovirus Genotyping Tool version 2.0 and BLAST classified two of the strains as GI.P2_GI.2 (GenBank accession number 4655/2015:MH327963) and the remaining as GII.P16_GII.13 (GenBank accession number 4657/2015:MH327962). The analysed samples were positive either for GI.P2_GI.2 or GII.P16_GII.13 and co-infection was not detected. BLAST search analysis of the RdRp sequence of the GII.P16_GII.13 strain demonstrated close identity >98% at nt level with the GII.P16_GII.13 strains detected in Germany in 2012 during a large scale gastroenteritis outbreak [7], with strains detected in sporadic cases of gastroenteritis in Italian children [6] and with strains associated also with sporadic cases of gastroenteritis in Russian Federation [14]. In the ORF2 region our strain shared higher identity (99% at nt level) not only with the strains identified in Germany, but also with strains from China implicated in sporadic cases and gastroenteritis outbreaks.

#### Environmental samples’ microbiological analysis

Concerning the 11th August sampling, all six water samples from the network supply were negative for *E. coli* and Enterococci. Three of the samples taken from points nearby the central square of coastal settlement, were found positive for total coliforms with simultaneous low levels of residual chlorine (0.13–0.15 mg/L).

In the water sample from the drilling on 11th of August (ground water well in a block of summer cottages in the village) high levels of Enterococci and coliforms were found, indicating possible faecal contamination.

Water sampling was repeated on 20th of August, after chlorination of the water network supply. All samples from the coastal settlement were negative for all bacterial indicators, whereas the residual chlorine was between 0.31 and 0.53 mg/L. Other chemical parameters tested where within the required limits of legislation.

### Further environmental investigation

Damages in two secondary distribution pipelines (pipeline breakage) in the coastal settlement before the onset of gastroenteritis cases were recorded by the municipality, which were repaired at the same day. We were informed that there was a common water tank for both the main village and the coastal settlement but different distribution pipelines, both primary and secondary. However, a thorough mapping of the water supply network was not available.

In the district, sewage is transferred with a network of pipes and pump stations to a municipal treatment plant, with the main pumping station located in coastal settlement central place. Several of the residents interviewed reported flooded wells of potable water and sewage overflow in the municipality system, perhaps due to the much higher catchment population during the high touristic season. However, there was no official confirmation of malfunction of the sewage drainage system during the whole investigation period. Furthermore, tourists and residents interviewed in the coastal settlement reported turbidity, coloration and unusual odour of drinking water.

#### Measures taken

In order to disinfect even for the most remote parts of the water supply network, the local authorities proceeded to an increase of the chlorine concentration, urged by the laboratory findings showing very low residual chloride levels along with the presence of total coliforms in the first water sample testing.

Guidance to the local authorities regarding the appropriate preventive measures was provided by the HCDCP, such as flushing of the pipelines and use of chlorination after water pipeline operations in order to protect pipeline water from possible contamination, especially during summer when the population is increased.

Furthermore, we recommended a mapping of the water distribution network pipeline to be available, routine implementation of control measures and systematic monitoring, training of staff as well as a complete incident registration record for future prompt and timely identification of any temporal and spatial association between gastroenteritis cases and damages in water distribution systems.

Finally, we urged the local authorities to include in their incident preparedness plans, the provision for the appropriate collection and storage of water samples during suspected waterborne disease outbreaks in order to assist the follow-up and the identification of the aetiological agent.

## Discussion

In August 2015, two distinct norovirus strains GI.P2_GI.2 and, the first reported in Greece, recombinant strain GII.P16_GII.13, were identified as the causative agents of a large gastroenteritis outbreak in Northern Greece, even though other norovirus strains or viral agents could not be excluded as causative agents of the outbreak since only three samples were genotyped. The investigation indicated that it was a large outbreak with a peak on 10 August, and a possible secondary person-to-person transmission after the 16th of August, which is the most touristic season for Greeks and foreign tourists. The tourist industry, including travel, lodging, food and night life services was the region's main source of revenue and thus the impact of the outbreak was great. The outbreak attracted media attention with substantial economic burden due to reservation cancellations and loss of vacation days for ill travellers.

The descriptive epidemiological data, the results of the environmental investigation as well as the results from the analytical study indicated as a possible source consumption of tap water. Furthermore, reports from tourists or residents in the coastal settlement of turbidity, coloration and unusual odour of the drinking water, in combination with the results of the microbiological and chemical analysis, further support this hypothesis. According to the revised strength of evidence classification of waterborne outbreaks of the Center for Diseases Control [15], this outbreak could be classified as class I.

The breakage of two pipelines in the coastal settlement just before the onset of cases, along with reports on flooded wells and sewage overflow, mainly around the central square, indicated a strong possibility of water contamination by sewage during the pipeline breakage. Incidents at the drinking-water distribution systems leading to a loss of water pressure and inadequate physical integrity of the distribution system have been shown to result in an increased risk of gastrointestinal illness [16, 17]. This association indicates that the pathogen contamination originates from an external source, as pathogens can be present in soil and water surrounding the drinking-water pipelines or in sewage, especially if sewage and drinking-water pipelines are close to one another [18] or even on the same level in the pipelines trench [17].

However, the local authorities did not consider the occurrence of gastroenteritis cases to be linked to the breakage, as they probably evaluated the incident of low risk for water contamination, based on the fact that the breakage had been repaired during the same day resulting in a rather short duration of low water pressure. Indeed, in situations of low-pressure incidents, the duration of the low pressure was one of the most important factors for the average risk of virus infection [18] and longer duration of water shutdown during break repairs has been shown to increase the risk of infection [16]. On the other hand, results from a recent study [17] indicated that the duration of low pressure may not be as important risk factor, as previously believed, in comparison with the condition of the surrounding of the water pipelines.

Norovirus outbreaks of waterborne origin are found in several studies and systematic reviews to be caused by GI only or both GI and GII strains over GII only strains, compared to foodborne ones, possibly due to an increased stability of GI strains in water compared to GII strains [10, 12, 19]. GII norovirus strains are more frequently implicated in foodborne, person-to-person and environmental outbreaks and, particularly GII.4, is more likely to be the causative strain in outbreaks that occurred in healthcare settings. However, a significantly higher proportion of water and foodborne outbreaks is associated with multiple strains (GI + GII) appearing simultaneously [10].

The simultaneous detection of both GI and GII norovirus genotypes in our investigation, in combination with the laboratory findings on the first water samples, strongly supports the hypothesis of water contamination by human sewage, which contain a mixture of viruses circulating in a given community and thus resulting in outbreaks with multiple strains; so while some exposed individuals may have had either innate or acquired resistance to some strains, they could have been susceptible to other strains [10, 19, 20]. Notably, early recognition of norovirus diversity in a cluster of patients, even when samples are tested negative for bacterial pathogens, parasites and other viruses could be used as a useful warning marker of waterborne contamination during an outbreak [21]. These findings contrast with norovirus person-to-person transmission occurring in settings such as hospitals or nursing homes, which most often involve a single genotype [22].

The potential of noroviruses to cause outbreaks is also driven from recombination events. Recombination is a driving force in norovirus evolution and is responsible for the emergence of novel variants, which potentially may cause outbreaks, since the ‘jumping evolution’ of recombinants permits norovirus to escape host immunity [23]. The investigated outbreak was attributed to two different norovirus strains. GI.P2_GI.2 strains that are relatively rarely reported [5], but they proved to be the causative agent for 23 linked point-source gastroenteritis outbreaks in Denmark in April 2016 [24]. The outbreaks occurred over the course of 1 week and norovirus GI.P2_GI.2 was detected to fresh green coral lettuce which was consumed by the patients. The second detected strain was the recombinant GII.P16_GII.13. The parental GII.P16_GII.16 norovirus was first detected in India and China in 2000 [25, 26], whereas GII.P13_GII.13 norovirus was reported for the first time in India in 2006 [27]. GII.P16_GII.13 noroviruses were already circulating in Italy in 2010, 2 years before the large-scale German outbreak [6]. In Greece, according to studies conducted by Ruether *et al*., the recombinant norovirus strain GII.P9_GII.6 was predominant and was circulating in the population of central Greece from 2006 to 2011 [9]. In a subsequent study, Ruether *et al.* described intergenogroup GII.P9_GI.7 and intergenotype GII.P9_GII.4 [28].

In the current investigation, although the epidemiological data strongly supported the waterborne origin of the outbreak, there was no laboratory confirmation by water analyses data for the pathogen detected to cases. The inadequate local laboratory capacity to examine the full spectrum of implicated pathogens, as well as the local authorities' unintentional disregard to collect water samples for virological testing, were the main reasons for the lack of virological testing of the water. The latter was mainly due to the over reliance on the absence of bacterial indicators during the routine water quality monitoring carried out for several months prior to this outbreak, as well as the absence of *E. coli* and Enterococci, as the basic bacterial indicators of faecal contamination in the first water sample testing. However, previous evidence showed that the commonly used bacterial indicators could be unreliable regarding viral contamination of water, since their survival is shorter than that of enteric viruses, especially norovirus and Hepatitis A virus. Thus, the presence of norovirus may not correlate with bacterial indicators of faecal contamination [29].

Unfortunately, during the current outbreak investigation the possibility of water contamination by sewage during the pipeline breakage could not be proved nor a definite conclusion of how the water was contaminated could be made. The absence of mapping of both the water distribution and sewage pipelines, the rather incomplete registration of the breakage incident and the absence of a thorough laboratory examination of water samples for the full spectrum of implicated pathogens were the basic limitations in order to prove the source of the outbreak.

In the presence of the aforementioned challenges we turned to the analytical study in order to reach to useful conclusions on the source of the outbreak. The purpose was to recommend measures for preventing future similar outbreaks. The cooperation with the local authorities was close and their prompt intervention for water hyperchlorination was crucial for preventing further transmission.

In conclusion, this outbreak investigation demonstrated the importance of application of a systematic and proactive approach for water safety in touristic areas. Development of a water safety plan following methods given by the World Health Organization is recommended for the municipality water as an effective way to ensure water safety [30]. Moreover, this outbreak investigation emphasises the need for implementing standard operational procedures and supporting mechanisms in multiple levels in order to reach to a comprehensive national surveillance system for viral gastroenteritis infections and outbreaks. Moreover, continuous molecular surveillance is required in order to identify timely the emergence of novel norovirus variants. In addition, enhancement of the laboratory investigation of waterborne outbreaks in the country with stimulation of diagnostic testing for viruses in water samples and implementation of a collaboration protocol between local authorities and reference laboratories, which would clearly define when water samples should be collected, how could they be stored and transferred for virological testing, even in the absence of bacterial indicators of faecal contamination in water samples, are essential.
